# The power features of Masseter muscle activity in tension-type and migraine without aura headache during open-close clench cycles

**DOI:** 10.7717/peerj.3556

**Published:** 2017-07-25

**Authors:** Behrouz Alizadeh Savareh, Ali Ghanjal, Azadeh Bashiri, Monireh Motaqi, Boshra Hatef

**Affiliations:** 1 Student Research Committee, School of Allied Medicine, Shahid Beheshti University of Medical Sciences, Tehran, Iran; 2 Health Management Research Center, Baqiyatallah University of Medical Sciences, Tehran, Iran; 3 Health Information Management Department, School of Allied Medical sciences, Tehran University of Medical Sciences, Tehran, Iran; 4 Physiotherapy Research Center, School of Rehabilitation, Shahid Beheshti University of Medical Sciences, Tehran, Iran; 5 Neuroscience Research Center, Baqiyatallah University of Medical Sciences, Tehran, Iran

**Keywords:** Power, EMG, Aura, Migraine, Headache, Masseter

## Abstract

**Introduction:**

Different types of headaches and TMJ click influence the masseter muscle activity. The aim of this study was to assess the trend of energy level of the electromyography (EMG) activity of the masseter muscle during open-close clench cycles in migraine without aura (MOA) and tension-type headache (TTH) with or without TMJ click.

**Methods:**

Twenty-five women with MOA and twenty four women with TTH participated in the study. They matched with 25 healthy subjects, in terms of class of occlusion and prevalence of temporomandibular joint (TMJ) with click. The EMG of both masseter muscles were recorded during open-close clench cycles at a rate of 80 cycles per minute for 15 seconds. The mouth opening was restricted to two centimeters by mandibular motion frame. Signal processing steps have been done on the EMG as: noise removing, smoothing, feature extraction, and statistical analyzing. The six statistical parameters of energy computed were mean, Variance, Skewness, Kurtosis, and first and second half energy over all signal energy.

**Results:**

A three-way ANOVA indicated that during all the cycles, the mean of energy was more and there was a delay in showing the peak of energy in the masseter of the left side with clicked TMJ in MOA group compared to the two other groups, while this pattern occurred inversely in the side with no-clicked TMJ (*P* < 0.009). The variation of energy was significantly less in MOA group compared to the two other groups in the no-clicked TMJ (*P* < 0.003). However, the proportion of the first or second part of signal energy to all energy showed that TTH group had less energy in the first part and more energy in the second part in comparison to the two other groups (*P* < 0.05).

**Conclusion:**

The study showed different changes in the energy distribution of masseter muscle activity during cycles in MOA and TTH. MOA, in contrast to TTH, had lateralization effect on EMG and interacted with TMJ click.

## Introduction

The generation of semi-automatic movements like chewing is in the central pattern generator (CPG). CPG is a group of neurons that are organized in the spinal cord to induce repeated movement. CPG is modulated by the supra-spinal descending pathway (Vogt, Pfeifer & Banzer, 2003). The proprioception afferents and pain projections also have an important role to modulate the CPG (Westberg, 1999). The projection of CPG and peripheral sensory afferents such as proprioception and pain are integrated to gamma motor neuron to control sensitivity of motor neuron (Capra, Hisley & Masri, 2007; Ellaway, Taylor & Durbaba, 2015). The change of motor unit activity is reflected and it is visible in some features of EMG (Farina & Holobar, 2016).

Some studies showed the cervico-cephalic reflexes are sensitized in headache due to central pain facilitation (Reshkova, Bogdanova & Milanov, 2015). Electromyography recording of muscles in headache patients especially in tension type headache, (TTH) showed increase of intensity of rest and duration of masseter muscle activity during chewing-like motion (Hatef et al., 2012; Jensen, Fuglsang-Frederiksen & Olesen, 1994; Shimada, Baad-Hansen & Svensson, 2015; Sohn, Choi & Jun, 2013). Some models had been proposed based on the results of many studies about interaction of pain and motor activity (Sessle et al., 2008). Modified pain adaptation model explains that the central motor command to control pattern of agonist and antagonist muscle activity during a rhythmic movement changes to limit the range of mobility and variation in movement (Minami et al., 2013; Peck, Murray & Gerzina, 2008).

It seems that several types of headaches influence motor planning differently. This means that whereas the regions of pain of TTH and migraine are very close to each other, the changes of intensity and duration of masseter muscle activity and responses to motor reflex were significantly different between them (Hatef et al., 2012; Jensen, Fuglsang-Frederiksen & Olesen, 1994; Proietti Cecchini et al., 2003). Previous studies had assessed the static or dynamic activity of muscles in headache and reported the mean of changes not the trend during muscular activity. This means that they did not evaluate the pattern of activity during a period of time. To evaluate the effect of pain and proprioception disturbance on the output of CPG in the EMG, the aim of this study was to investigate the effect of TTH and migraine without aura (MOA) headache on the pattern of the energy distribution of the masseter muscle activity during repeated open-close-clench cyclesinteracted with TMJ click.

## Methods

### Participants

A total of 49 adult females with primary headaches participated in this case-control study. All the participants were clearly informed about the nature of the study by signing approvalforms given by the medical ethical committee of rehabilitationInstitute of Tehran University of Medical Sciences. A neurologist diagnosed the patients as having a headache based on the international guidelines for the classification of headache disorders, (beta version) (Headache Classification Committee of the International Headache Society (IHS), 2013), and were categorized into two groups of TTH (25 patients) and MOA (24 patients). The control group included twenty five healthy participants having no defined sign of headache. All the participants were clearly informed about the nature of the study by signing approval forms given by Tehran University of Medical Science.

All of the participants had the following characteristics: (1) No surgery or trauma in the cranium and spine regions; (2) No history of orthodontic or missing teeth, except for the second and/or the third molars; (3) No temporomandibular disorders.

The occlusion class and the presence of TMJ with sounds were assessed to control and match their distribution. The class of occlusion was determined based on Angle’s classification method in which the relationship between the upper and lower first molar and upper and lower canine were considered. Bilateral posterior palpation of TMJ through the external auditory meatus was performed by fingertips to detect the TMJ with sounds (click or crepitation). The participants were asked to open and close their jaw in a functional range (2–3 cm). If a participant or examiner sensed joints with repeatable sounds or abnormal grinding, those joints were considered as having click (Pertes & Gross, 1995). The patients were asked about some characteristics of headache such as the frequency of headache per month and years of headache and they were excluded if they reported any headaches three days before the test (Hatef et al., 2007; Hatef et al., 2012).

### EMG recording

Firstly, the skin under the electrodes was cleaned by alcohol. Bipolar surface electrodes (Ag/Ag-Cl) with a 20 mm inter-electrode distance were filled by gel and were placed on a line parallel to the masseter muscle fibers. Electrode impedance was under 5 kΩ (Hatef et al., 2007). The ground electrode was attached around the right wrist. The EMG signal was recorded by Premiere model, Medelec, with a CMRR >100 db.

### Procedure

The participants were sitting on a chair in the erect position to maintain their head in the Frankfort horizontal position. Mandibular motion in the sagittal plane was limited by the mandibular motion frame which was fixed on mandible and external frame which limited the opening of the mouth to two centimeters (Hatef et al., 2007). The participants were first asked to relax their jaw followed by completing the cycles of jaw opening, closing and clenching the teeth at the rate of 80 cycles per minute monitored by voice metronome. The EMG signals of both masseter muscles were recorded during the motion (Hatef et al., 2007).

### Data processing

The data was saved digitally. The offline sequence of noise removing, smoothing, feature extraction and statistical analyzing had been performed. The first three steps were implemented in MATLAB and SPSS version 21 was used to analyze the data.

### Noise removing

Noise removing from EMG signals must be done by considering the signal properties. EMG is a signal in which various noises and multiple sources of noises appear (Kale & Dudul, 2009; Katariya & Devasahayam, 2006). Noise removing has been done by two steps as follows:

#### Removing unwanted frequencies

EMG has a frequency range between 10 and 500 Hz, so a high-pass filter was used to remove the frequencies below the threshold by applying a Butterworth filter which creates the pass band as flat as possible (Thede, 2005). Based on Nyquist theory, using the sampling rate of 1024 Hz in data collection, there is no need to use a low-pass filter.

#### Suppressing sparse areas of EMG signal

**Figure 1:**
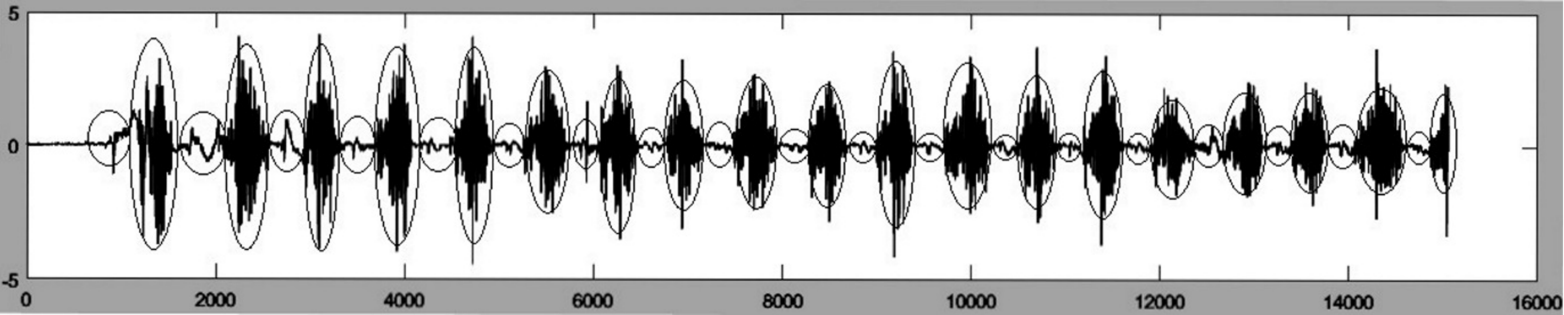
EMG of a sample masseter muscle activity.

EMG of masseter in sequential jaw pressing has a semi-regular pattern of sequential activity and silence (Fig. 1). The silence area (small ellipses) where noises exist without a real activity must be removed. One of the methods that can be used in this situation is Overlapping Group Sparse Denoising (OGSD) that is based on convex optimization and non- convex regularization. The method acts using the combination of convex and non-convex optimization and has the benefits of both approaches simultaneously (Chen & Selesnick, 2014).

In order to use OGSD for noise removing, the signal must be in the form of Group—Sparse in which the values of the signals tend to create separate clusters, without predefined clear boundaries and also their start and finish points are not regular (Chen & Selesnick, 2014). As shown in Fig. 1, EMG signal of masseter muscle in jaw pressing has such properties. In this study, OGSD was used to remove sparse area noises.

### Smoothing

Smoothing is one of the most important procedures used in preprocessing phase of signal processing to increase the signal-to-noise ratio (SNR) of any signal (Jo et al., 2007) and EMG. Smoothing is useful when some unwanted spikes have appeared as the result of any body movement during EMG recording. To overcome these spikes a smoothing step was applied on signals by Savitzky-Golay Filtering (Orfanidis, 1995). This method works by applying a cubic Savitzky-Golay filter on signal which is suitable for EMG signal smoothing.

### Feature extraction:

Waveform length feature extraction was used to feature extraction from the signals (Chan & Green, 2007). This method is based on getting feature from signal by sliding a window on it to calculate the energy of signal (Code Block 1).

for i = 1:numwin

% sliding over all of windows

curwin = x (st:en,:).*datawin;

% applying windowing effect on the cut part of signal (e.g., hamming window)

feat(i,:) = sum(abs(curwin.*curwin));

% extracting feature

st = st + wininc;

% setting new start point

en = en + wininc;

% setting a new end point

end

Code Block 1. Algorithm of WLFeat

In this study, a logarithmic version of convolution was used to feature extraction (Code Block 2). Customized logarithmic feature extraction was used to overcome noise impact on energy calculation.

fori = 1:numwin

% sliding over all of windows

curwin = x(st:en,:).*datawin;

% applying windowing effect on the cut part of the signal (e.g., hamming window)

feat(i,:) = log 10(sum(abs(curwin.*curwin)));

% extracting logarithmic feature

st = st + wininc;

% setting new start point

en = en + wininc;

% setting a new end point

end

Code Block 2. Algorithm of customized WLFeat

### Statistical analysis

To compare the baseline characteristics such as the age between three groups, year and the frequency of headache between TTH and MOA groups, the Mann–Whitney test was used. Chi-square test was done to compare the distribution of the class of occlusion and existence of click in TMJ. The feature extraction was done for all the signals, and then to compare the three groups, six parameters of energy were computed on (Table 1). Mean, Variance, Skewness, Kurtosis, and first and second half energy over all signal energy were calculated for the three groups of signals on both left and right sides.

**Table 1:** Six parameters of energy.

Parameter	Description	Interpretation
Mean (1st moment)	Estimating the value around which central clustering occurs	High value indicates high energy in signal overall.
Variance (2nd moment)	The measurement of the spread between the numbers in a data set. The variance measures how far each number in the set is from the mean.	High value indicates high difference of energy level.
Skewness (3rd moment)	Characterizing the degree of asymmetry of a distribution around its mean	Positive skewness indicates that the data are positively skewed or skewed right, meaning that the right tail of the distribution is longer than the left (decreasing energy by time) and vice versa.
Kurtosis (4th moment)	Measuring the relative peakedness or flatness of a distribution	High kurtosis value shows the concentration of activity in the middle of the signal to the tails.
First half energy over all signal energy	The sum of signal activity in the first half of signal over the whole signal	The high value of the parameters shows a high portion of signal energy occurred in the first part of the signal.
Second half energy over all signal energy	The sum of signal activity in the second half of signal over the whole signal	The high value of the parameters shows a high portion of signal energy occurred in the second part of the signal.

It was useful to characterize the set by a few numbers related to its moments below general relation for n’th moments (Press et al., 2015).

\begin{eqnarray*}{\mathrm{\mu }}_{n}=\mathrm{E}[(X-\mathrm{E}[X])^{n}]. \end{eqnarray*}

Clicked TMJ and side were considered as factors in addition to the three groups. Then, the cases were divided into two categories of clicked or no-clicked TMJ. Another categorization was based on the masseter of left or right side.

**Figure 2:**
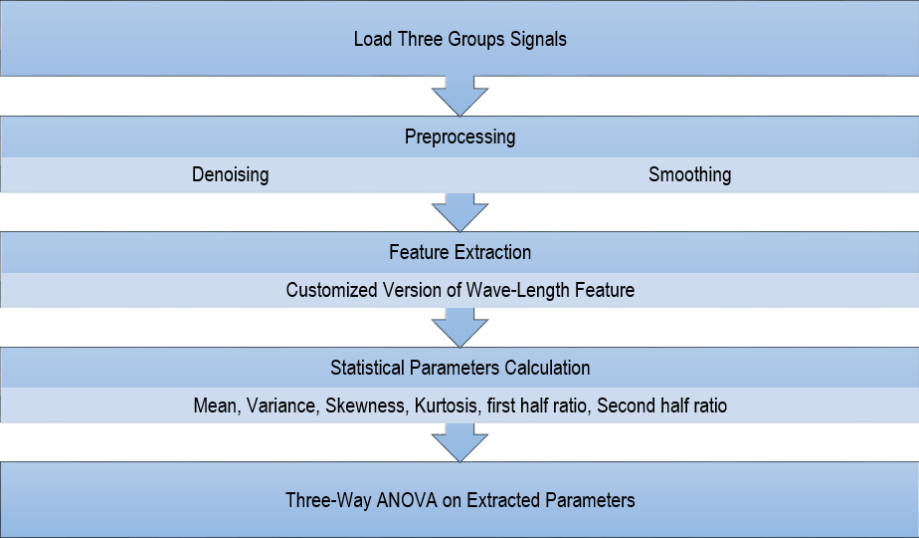
Flowchart of all the conducted steps.

Subsequently, a three-way ANOVA analysis (Control/Migraine/Tension Headache, left/right, and TMJ with/without click) was conducted upon the above parameters since they had a normal distribution. The flow chart of all the steps is shown in Fig. 2. A *P*-value less than 0.05 was considered as significant.

## Results

Table 2 gives a comparative statistics of the demographic and clinical characteristics for the participants of the three groups. The prevalence of occlusion type classes and TMJ with click (click or crepitus) category were not significantly different among the three groups. The patients of TTH group were significantly younger than the two other groups. The mean of the frequency of headache was around nine headaches per month in both headache groups but the years of headache in MOA group were twice as more as TTH group.

**Table 2:** The demographic and clinical characteristics of the participants.

	Control G (*N* = 25)	Migraine without aura G (*N* = 24)	Tension-Type headache G (*N* = 25)	*P*-value
Age (mean ± SD)	26.3 ± 9.8	28 ± 10.5	22.6 ± 7.1	0.008^a^
Type of occlusion (percentage of class I/II/III)	54.5/31.8/13.6	37.5/41.6/20.8	64/24/12	0.38
Clicked TMJ (% )	29.6	36	42.3	0.79
Years of headache (mean ± SD)		10.1 ± 7	5.2 ± 3	0.001
Frequency of headache( times in month)		9 ± 6	9.5 ± 7	0.74

**Notes.**

a A significant difference between TTH and MOA and between TTH and control group based on 4 Mann–Whitney test.

Tables 3 and 4 represent the descriptive statistics of EMG variables (mean, lower and upper band of 95% confidence interval of the mean) of the three groups (Control, MOA and TTH groups), two sides (right and left) with or without clicked TMJ.

**Table 3:** Rrepresents the mean and the upper and lower band of 95% confidence interval of variables in the three groups with clicked or no-clicked TMJ of the right side.

Variables	Right						
	Group	Clicked TMJ			No-clicked TMJ		
		95% Confidence Interval			95% Confidence Interval		
		Mean	Lower	Upper	Mean	Lower	Upper
Mean	Control	−2.98	−3.4	−2.5	−2.81	−3	−2.5
	MOA	−2.73	−3.1	−2.3	−2.81	−3	−2.5
	TTH	−2.64	−2.9	−2.2	−2.66	−2.9	−2.3
Variation	Control	4.038	3.337	4.740	4.513	4.076	4.950
	MOA	4.352	3.733	4.970	4.521	4.042	5
	TTH	4.933	4.346	5.520	4.507	4.028	4.986
Kurtosis	Control	2.126	1.316	2.936	1.922	1.417	2.427
	MOA	2.079	1.365	2.794	2.015	1.462	2.568
	TTH	1.486	.809	2.164	1.766	1.213	2.319
Skewness	Control	.880	.403	1.357	.725	.427	1.022
	MOA	.672	.251	1.092	.744	.418	1.070
	TTH	.525	.126	.924	.559	.233	.885
Mean of first part	Control	.571	.523	.620	.548	.518	.578
	MOA	.559	.517	.602	.534	.501	.567
	TTH	.520	.480	.561	.498	.465	.531
Mean of second part	Control	.429	.381	.477	.452	.422	.482
	MOA	.441	.398	.483	.466	.433	.499
	TTH	.480	.439	.520	.502	.469	.535

**Table 4:** Representation the mean, the upper and lower band of 95% confidence interval of variables in the three groups with clicked or no-clicked TMJ of the left side.

Variables	Left						
	Group	Clicked TMJ			No-clicked TMJ		
		95% Confidence Interval			95% Confidence Interval		
		Mean	Lower	Upper	Mean	Lower	Upper
Mean	Control	−2.946	−3.296	−2.596	−2.381	−2.677	−2.085
	MOA	−1.900	−2.291	−1.508	−3.203	−3.572	−2.834
	TTH	−2.920	−3.339	−2.502	−2.626	−2.912	−2.340
Variation	Control	4.295	3.709	4.882	5.123	4.628	5.619
	MOA	4.665	4.010	5.321	3.685	3.067	4.304
	TTH	4.201	3.500	4.903	4.312	3.833	4.791
Kurtosis	Control	2.077	1.399	2.754	1.301	.728	1.874
	MOA	1.660	.902	2.417	2.353	1.595	3.110
	TTH	2.133	1.323	2.943	1.824	.454	1.251
Skewness	Control	.853	.454	1.252	.265	-.072	.602
	MOA	−.260	−.706	.187	1.031	.585	1.478
	TTH	.837	.360	1.314	.590	.265	.916
Mean of first part	Control	.524	.484	.565	.540	.506	.575
	MOA	.544	.499	.589	.529	.486	.571
	TTH	.498	.450	.546	.528	.494	.562
Mean of second part	Control	.476	.435	.516	.460	.425	.494
	MOA	.456	.411	.501	.471	.429	.514
	TTH	.502	.454	.550	.472	.438	.506

**Figure 3:**
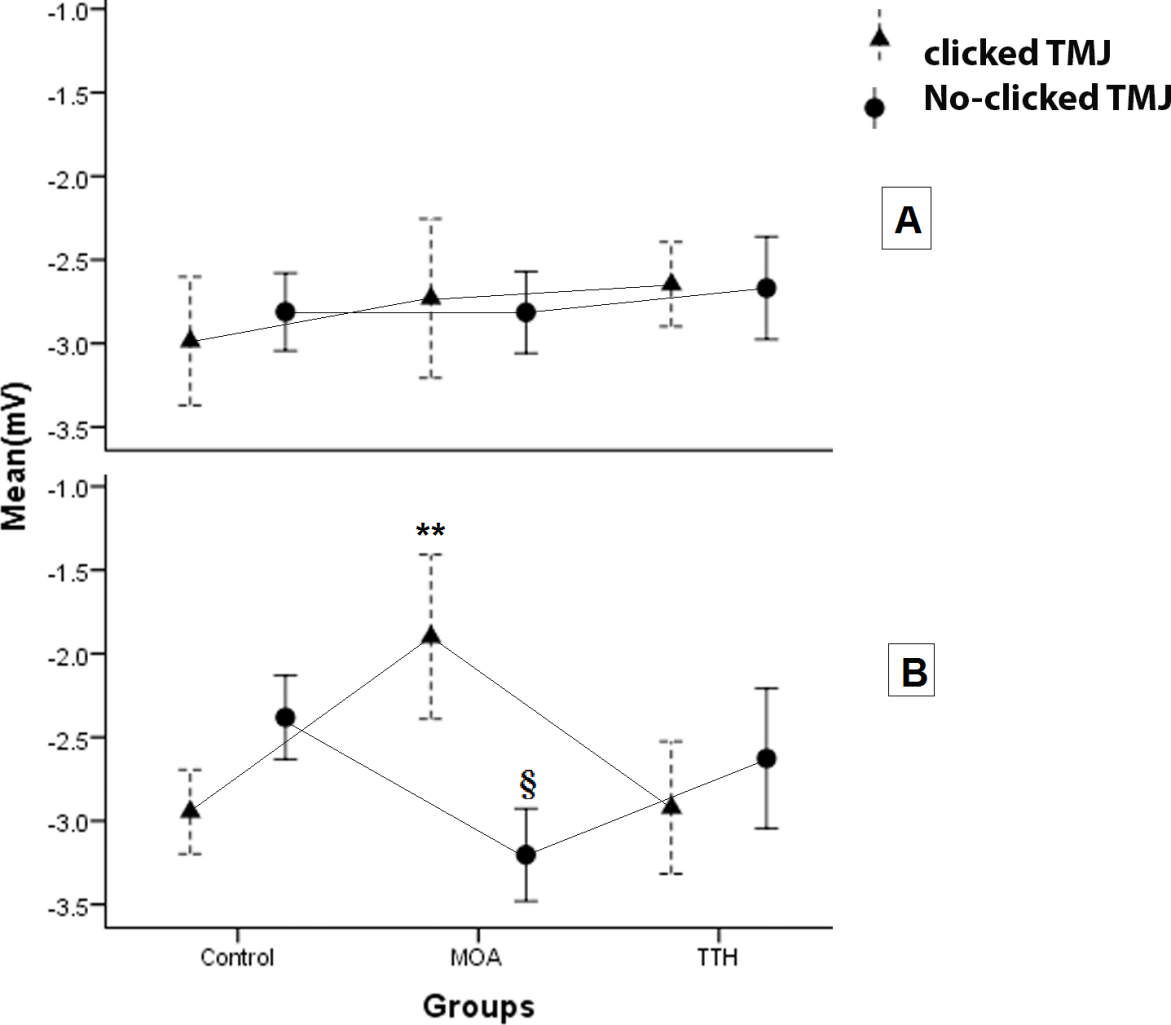
The plots show the mean of the energy of masseter activity during the cycles in the two sides of the three groups with or without clicked TMJ. ^∗∗^: A significant difference was seen in the left side among MOA group and the two other groups in clicked TMJ (*P*-value < 0.002). §: A significant difference was seen in left side among the MOA group and the two other groups in no-clicked TMJ (*P*-value < 0.02).

**Figure 4:**
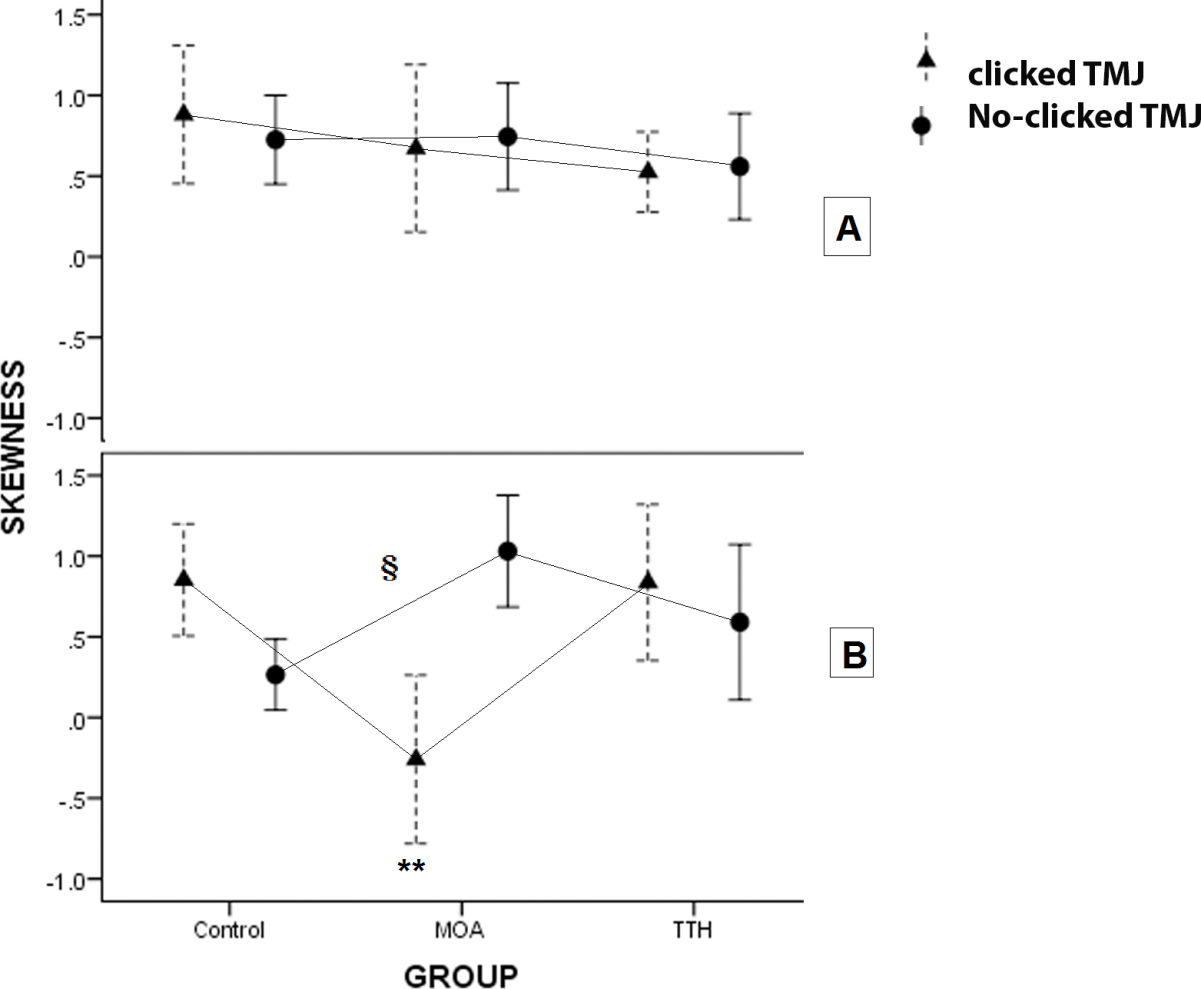
The plots show the skewness of energy of masseter activity during the cycles in the two sides of the three groups with clicked and no-clicked TMJ. **: A significant difference was seen in the left side among MOA group and the two other groups in clicked TMJ (*P*-value < 0.002). §: There was a significant difference in the left side between MOA group and the control group in no-clicked TMJ (*P*-value < 0.002).

The data were distributed normally and the equal variances were assumed. The results of the three-way ANOVA and the following Bonferroni collection test evaluating the interaction effect of groups, side of EMG recording and the existence of clicked TMJ as main factors were presented in Table 5. When the time of cycles was divided into two parts, the energy of muscle activity can be compared between the main factors (group, TMJ click and side) in the first and last part. The power of masseter activity of TTH group had less activity in the first part to all the cycles and more power of activity in the second part to all than the two other groups independently to side and TMJ click (*P*-value <  0.04). In the left side, the masseter muscle activity of MOA groupshowed a higher mean power of activity than the two other groups in the clicked TMJ (*P*-value <  0.002) and less mean power than that in the no-clicked TMJ (*P*-value <  0.02) (Fig. 3). Variation from mean power significantly decreased in the MOA group than the control group in both sides in the no-clicked TMJ. Skewness which showed the pattern of power during cycles was affected by main factors. The results showed that the Skewness of the left masseter muscle in MOA group was less than the two other groups in the clicked TMJ (*P*-value <  0.002) and it was more only in the control group in the no-clicked TMJ (*P*-value <  0.009) (Fig. 4). Kurtosis of masseter activity did not show a significant difference between the groups and sides and the existence of TMJ click (*P*-value >  0.2).

**Table 5:** *P*-value of the three-way ANOVA.

Variables	Groups	Side	TMJ click	Groups × Side	Groups × TMJ click	Side × TMJ click	Groups × Side × TMJ click
Mean	0.62	0.26	0.53	0.26	0.000	0.39	**0.002^a^**
Variation	0.59	0.56	0.86	0.07	**0.02^b^**	0.79	0.09
Skewness	0.64	0.25	0.55	0.17	0.001	0.46	**0.008^c^**
Kurtosis	0.66	0.95	0.74	0.34	0.24	0.73	0.29
Mean of the first part	**0.03^d^**	0.33	0.57	0.53	0.68	0.14	0.76
Mean of the second part	**0.03^d^**	0.33	0.57	0.53	0.68	0.14	0.76

**Notes.**

a A significant difference among MOA and the two other groups in the left masseter muscle in TMJ with and without click.

b A significant difference between MOA and the control group in the left masseter muscle of no-clicked TMJ.

c A significant difference among MOA and the two other groups in the left masseter muscle in clicked TMJ and between MOA and the control group in the left masseter muscle in the no-clicked TMJ.

d A significant difference among TTH and the two other groups.

## Discussion and Conclusion

The results showed that the effect of the type of headache on the distribution of the energy of EMG activity was different in masseter muscle during the short time of chewing-like movement. The comparison of the MOA group to other groups showed that the greater the mean of the power with delayed height of energy of the left masseter muscle during cycles in the clicked TMJ and inverted pattern in the no-clicked TMJ. On one side, the variation of energy was significantly less in MOA group than the two other groups in the no-clicked TMJ. On the other side, the relative first or second time parts to all time showed that the TTH group had less energy in the first part and more energy in the second part in comparison with the other groups. The TMJ click had a critical effect on the change of muscle activity in the MOA group. Previous studies revealed that TMJ click changes the masseter muscle timing during chewing-like movement significantly (Hatef et al., 2007). The finding showed that the differences were more on the left side. Some other potential conclusions can be presented. It might be related to right-hand participants or the MOA may also have a lateralization effect. The left side might be affected by MOA greater than the right side. It was not clearwhy the TMJ click inverted the distribution of power of masseter muscle activity in the MOA subjects during chewing-like movement. However, the difference between TTH and other groups was not related to the side of recording and the existence of TMJ click.

The experimental studies showed that the pain afferents and CPG inputs are projected and integrated to interneurons. A premotor neuron of caudaltrigeminal nucleus modulates the gama motor neuron and alpha motor neuron activity (Capra, Hisley & Masri, 2007; Westberg, 1999). Nevertheless, the pain descending pathway from anterior cingulate cortex (ACC)- Periaqueductal gray matter (PAG)- Rostral ventromedial medulla (RVM)- dorsal horn (DH) axis and the projection of dorso-caudal of ACC to motor regions reveal extensive evidence to show the significant role of pain to modulate motor activity (Rainville, 2002). Previous studies demonstrated that headaches affect the timing and intensity of masseter muscle activity (Burnett et al., 2000; Hatef et al., 2012; Jensen, Fuglsang-Frederiksen & Olesen, 1994). Some of them compared the effect of two tension-type and migraine headache on EMG. They found that the level of EMG of temporalis muscle at rest or frontalis muscle at mental work in Tension-type were higher than migraine headache (Sandrini et al., 1994). The timing pattern of the masseter muscle activity during chewing showed more changes in TTH than MOA patients (Hatef et al., 2012). The evaluation of reflexes such as terigemocervical or blink reflex showed that chronic TTH and migraine had more influence on them than acute TTH (Nardone & Tezzon, 2003a; Nardone & Tezzon, 2003b; Proietti Cecchini et al., 2003). When the headache became chronic, the pain matrix included supra-spinal regions especially anterior cingulate cortex was sensitized to pain stimulus and showed more effect in motor planning (Millan, 2002).

In the present study, the years of headache in the MOA group were twice as much as the TTH group. Also, some changes in MOA were seen which did not occur in the TTH group. However, muscle pain afferents, type III fibers, are involved more in TTH than in migraines (De Tommaso & Fernandez-de Las-Penas, 2016) and type III fiber afferents in muscle had a more significant effect on the sensitivity of the motor neuron and its muscular reflex (Schomburg et al., 2012). Some of the findings are defensible because the involvement of type III fibers is more in TTH than in migraines. TTH showed that the level of the energy of masseter activity was lower in earlier cycles and it became higher in later cycles in comparison to the two other groups. The findings revealed the new and different aspects of the modulation of TTH and migraine headaches on the activity of masseter muscle during repeated jaw motion like chewing. The change of energy rising in MOA was more complex. There was no significant difference in the dominance of headache side in the MOA patients by asking about headache occurrence lately, although there was a significant difference in the pattern of energy changing during the cycles in left side with or without TMJ click. Furthermore, there is an open question about the lateralization effect of migraine on the masseter muscle behavior which did not occur in TTH.

The sensory inputs such as proprioception from muscles and most closely joints implicated in controlling the movement interacted with CPG (Prochazka & Ellaway, 2012). The disturbance of the joint kinematics could change the proprioception inputs and CPG pattern to activate the muscles (Martin et al., 2012). Click in TMJ, as a sign of jaw dysfunction, can affect jaw CPG and masseter muscle activity. The current study confirmed this sequence which showed the existence of TMJ click interacted with the effect of headache on the mean and skewness of masseter muscle power during the chewing-like movement.

##  Supplemental Information

10.7717/peerj.3556/supp-1 Supplemental Information 1 Supplementary MATLAB codes

## Additional Information and Declarations

### Data Availability

The following information was supplied regarding data availability: The raw data has been supplied as a Supplementary File.
